# Deep learning infused SIRVD model for COVID-19 prediction: XGBoost-SIRVD-LSTM approach

**DOI:** 10.3389/fmed.2024.1427239

**Published:** 2024-09-03

**Authors:** Hisham Alkhalefah, D. Preethi, Neelu Khare, Mustufa Haider Abidi, Usama Umer

**Affiliations:** ^1^Advanced Manufacturing Institute, King Saud University, Riyadh, Saudi Arabia; ^2^Department of Computer Science and Engineering, Faculty of Engineering and Technology, SRM Institute of Science and Technology, Chennai, Tamil Nadu, India; ^3^School of Computer Science Engineering and Information Systems (SCORE), Vellore Institute of Technology, Vellore, Tamil Nadu, India

**Keywords:** deep learning, extreme gradient boosting (XGBoost), susceptible-infected-recovered-vaccination-deceased (SIRVD), long short-term memory (LSTM), feature selection, COVID-19, prediction

## Abstract

The global impact of the ongoing COVID-19 pandemic, while somewhat contained, remains a critical challenge that has tested the resilience of humanity. Accurate and timely prediction of COVID-19 transmission dynamics and future trends is essential for informed decision-making in public health. Deep learning and mathematical models have emerged as promising tools, yet concerns regarding accuracy persist. This research suggests a novel model for forecasting the COVID-19’s future trajectory. The model combines the benefits of machine learning models and mathematical models. The SIRVD model, a mathematical based model that depicts the reach of the infection via population, serves as basis for the proposed model. A deep prediction model for COVID-19 using XGBoost-SIRVD-LSTM is presented. The suggested approach combines Susceptible-Infected-Recovered-Vaccinated-Deceased (SIRVD), and a deep learning model, which includes Long Short-Term Memory (LSTM) and other prediction models, including feature selection using XGBoost method. The model keeps track of changes in each group’s membership over time. To increase the SIRVD model’s accuracy, machine learning is applied. The key properties for forecasting the spread of the infection are found using a method called feature selection. Then, in order to learn from these features and create predictions, a model involving deep learning is applied. The performance of the model proposed was assessed with prediction metrics such as *R*^2^, root mean square error (RMSE), mean absolute percentage error (MAPE), and normalized root mean square error (NRMSE). The results are also validated to those of other prediction models. The empirical results show that the suggested model outperforms similar models. Findings suggest its potential as a valuable tool for pandemic management and public health decision-making.

## 1 Introduction

The COVID-19 epidemic has presented a serious threat to civilization worldwide. The virus has killed millions of people and spread quickly. World Health Organization (WHO) at the end of 2019 announced COVID-19 as global epidemic disease, since its outbreak worldwide. As of November 6, 2023, reported by WHO, there are 775,335,916 confirmed cases and 7,045,569 deaths worldwide (1). Based on WHO data, 13.59bn vaccine doses have been given as of May 2, 2024. COVID-19 immensely affected daily life, health, and the economy at the global level. Governments and public health experts have put in place a number of strategies to prevent the epidemic, including social isolation, mask use, and vaccine. However, the transmission of the virus has not totally been halted by these precautions. Predicting how the pandemic will develop in the future is one of the difficulties in combatting COVID-19. This is significant for various reasons. First, it can assist governments and public health experts in making choices regarding the distribution of resources and pandemic response. Second, it can assist organizations and people in making decisions regarding how to run and safeguard themselves. The upcoming course of COVID-19 transmission is forecasted using various techniques. Making use of mathematical models is one strategy. The transmission of the virus and its effects on various populations can be predicted using mathematical models. Mathematical modeling is a crucial device for analyzing epidemic infectious diseases, presented in 1927 by Kermack (2). Since the outbreak of the pandemic, various mathematical models have been employed in predicting the diseases, which are epidemic. The widely used mathematical models include SIR (3), which assesses susceptible, infected, and recovered rates (4), and SEIR (5), which evaluates based on susceptible, exposed, infected, and recovered rates. Furthermore, most of the research studies are the enhanced models derived from these two models. However, using mathematical models can be challenging and complex.

Machine learning is a different strategy for forecasting COVID-19’s future trajectory. Machine learning, a form of artificial intelligence, possesses the ability to gain information from data and produce predictions. The efficacy of models involving machine learning in predicting transmission of various illnesses, including influenza, has been established through empirical evidence. Many studies are available on predicting and transmitting the virus’s spread (6).

This paper introduces a novel deep learning model named Extreme Gradient Boosting-Susceptible-Infected-Recovered-Vaccinated-Deceased-Long Short-Term Memory (XGBoost-SIRVD-LSTM), which is designed to forecast the quantity of COVID-19 cases. The suggested XGBoost-SIRVD-LSTM model operates in four stages: (1) Data pre-processing, (2) XGBoost feature importance score feature selection, (3) SIRVD epidemic model design, and (4) LSTM prediction. The suggested model is tested using datasets from John Hopkins University’s CSSE (7) and Our World in Data (8). The dataset is first pre-processed using the min-max normalization technique. Second, the XGBoost is used for feature selection, which is done using the feature importance score. Finally, the optimal features are supplied into the SIRVD model to estimate the COVID-19 transmission with respect to time. Finally, the LSTM model is applied to the dataset for disease prediction. The empirical results suggest that the suggested model exhibits superior performance in relation to accuracy for predicting outcomes compared to alternative deep learning models.

The following are the research study’s contributions:

- In this study, we introduce a deep learning model that utilizes XGBoost-SIRVD-LSTM model to predict COVID-19 infection cases.
- The outcomes of the suggested model assessed in comparison with existing deep learning models and utilizing performance measures for prediction.

The remaining sections of the paper are structured as follows: Section 2 presents a summary of the current body of literature. Section 3 delves into background information of the techniques employed in the proposed model. Section 4 outlines the methodology proposed in detail. Section 5 explores the dataset, presents the experimental results, and includes a comparative analysis with other models.

## 2 Literature review

This section elaborates on numerous models for COVID-19 prediction found in the literature. A standard SIR model for predicting COVID-19 pandemic progression was proposed in Kartono et al. (9). The model was tested using the most recent confirmed cases from the WHO dashboard. The authors used this approach to forecast instances in Singapore, Saudi Arabia, Indonesia, and the Philippines. In their study, Kumar et al. (10) employed recurrent neural network (RNN) models, including gated recurrent unit (GRU) and LSTM cells, to predict the future patterns of COVID-19 cases. The researchers utilized the publicly accessible COVID-19 dataset from Johns Hopkins University and emphasized the importance of factors such as age, population density, healthcare infrastructure, and disease-prevention efforts in the rapid progression of the COVID-19 outbreak. To analyze the COVID-19 pandemic, the study conducted exploratory data analysis using machine-learning techniques, followed by the implementation of the SIR model (11). The most popular John Hopkins dataset for COVID-19 was used for experiments, with just data from the Kingdom of Saudi Arabia used to forecast instances. The researchers analyzed three possibilities for anticipating the progression of the outbreak and its possible resolution, namely new medicine, lockdowns, and no actions. The simulation results demonstrate that interventions such as new drugs and lockdowns outperform no-action scenarios. To forecast the COVID-19 instances, the MLP with feature selection (MLPFS) classification model was presented (12). This study was based on the characteristics and symptoms of Electronic Medical Records (EMR) patients. Three separate datasets and eight alternative models were utilized to evaluate the provided model. According to the experimental findings, the suggested MLPFS outperformed the other seven models chosen for comparison in terms of accuracy indicators, extracted number of features, and time required to implement the model. The SIRVD model, an extension of classic epidemiological models, incorporates vaccination and time-dependent fatality rates (13). Analyzing exact solutions and approximations, it reveals crucial insights into epidemic dynamics, offering benchmarks for numerical simulations. By applying analytical approximations, particularly effective for low cumulative infection rates, it elucidates the impact of vaccination and time-varying fatality rates, enabling precise parameter extraction from COVID-19 data, essential for pandemic management. Babaei et al. (14) explores integrability conditions and exact analytical solutions for the SIRV model, crucial for understanding COVID-19 dynamics, using a partial Hamiltonian approach. Analyzing two cases based on model parameters and considering different phase spaces, it provides insights into the dynamics of susceptible, infected, recovered, and vaccinated populations over time through graphical representations. Federico (15) addresses an optimal vaccination strategy within an SIRS compartmental model, aiming to minimize social and economic costs while reducing susceptibility. Theoretical contributions include a non-smooth verification theorem and conditions for well-posed closed-loop equations, while numerical implementations highlight the effectiveness of vaccination policies in long-term infection control, particularly with low reproduction and reinfection rates.

In an another study, researchers suggested a three-stage COVID-19 prediction, namely pre-processing, feature selection, and classification (16). Wrapper-based feature selection using Recursive Feature Extraction and embedded-based feature selection using Extra Tree Classifier were the two methods used. The naive bayes and restricted Boltzmann Machine models were employed for classification. The proposed approach was implemented using WHO data. According to the authors, the model worked well and produced better prediction results with feature selection than models without feature selection. In their previous work, the researchers put forth COVID-19 prediction models utilizing Susceptible_Infected_Recovered (SIR) and Susceptible_Exposed_Infected_Quarantined_Recovered (SEIQR) epidemic models for several countries, including Australia, United Kingdom, and Italy (3). To enhance parameters in these epidemic models (L-BFGS-B), they employed optimization algorithms such as Conjugate Gradient (CG), Nelder–Mead, restricted memory bound constrained, and the Broyden-Fletcher-Goldfarb-Shanno (BFGS). The performance of these two models was compared to the performance of two machine learning methods, prophet and logistic function. The authors discovered that the prophet model outperformed the logistic function and provided a superior prediction model for Italy and the United Kingdom than for Australia. The prediction accuracy was significantly increased once the models such as SIR and SEIQR were optimized. In their findings, the authors observed that the prophet model demonstrated superior performance compared to the logistic function, particularly in predicting the COVID-19 trends for United Kingdom and Italy, while its performance in the case of Australia was relatively less favorable. The accuracy of predictions was notably improved by optimizing the SIR and SEIQR models. In a separate study conducted by the authors of Chandra et al. (17), deep learning-based LSTM models were explored for predicting the future trajectory of COVID-19 in specific Indian states that experienced a high incidence of the disease. Various LSTM models, including LSTM, bidirectional, and encoder-decoder models, were developed for disease spread prediction. The authors highlighted that the encoder-decoder LSTM model exhibited superior prediction accuracy compared to other models. In Alassafi et al. (18), a comparison study was undertaken to assess the efficacy of RNN and LSTM models in predicting the spread of the coronavirus. The dataset utilized for this analysis consisted of data from Malaysia, Morocco, and Saudi Arabia, sourced from the European Center for Disease Prevention and Control. The authors examined the models’ effectiveness in predicting positive cases, recoveries, and COVID-19-related mortality rates. Also, estimating the potential quantity of cases over the next 7 days. Another research study (19) proposed an XGBoost-DNN classifier model for detecting network intrusions. The model employed XGBoost feature importance scores to select relevant features and utilized DNN for classification of network intrusions.

In a separate study, researchers introduced a feature selection based on ensemble approach with LSTM for network intrusion classification (20). Their method aimed to improve the accuracy of network invasion detection by utilizing LSTM along with ensemble-based feature selection. Youssef et al. (4) employed the SEIQR model and utilized real data of Saudi Arabia for predicting the transmission of COVID-19 cases. The results demonstrated the efficiency of the model suggested in analyzing epidemic spread, thus providing a basis for framing effective government policies.

The COVID-19 pandemic has significantly accelerated research on the development of predictive models for the pandemic’s future trajectory. Numerous models, including mathematical, machine learning, and hybrid models, have been put forth. The propagation of the virus can be simulated and the effects on various populations can be predicted using mathematical models that are based on epidemiological principles. However, using mathematical models can be challenging and complex. An artificial intelligence that can learn from data and predict the future is known as a machine-learning model. It has been demonstrated that machine-learning models are useful for forecasting the spread of other diseases, such as influenza. Nevertheless, machine-learning models can often rely heavily on the specific data they were trained on, resulting in potential challenges when attempting to generalize to new data. To overcome these limitations, hybrid models (21–23) merge the advantages of both mathematical models and machine learning approaches. By combining these two techniques, hybrid models have the potential to offer greater precision and accuracy compared to using either method in isolation. However, the development of hybrid models can be intricate and pose significant challenges. Despite the extensive research conducted thus far, there remains a need for more precise and reliable models to effectively forecast the future trajectory of COVID-19. Thus, this study endeavors to fill this research void by proposing a novel model that leverages the strengths of both mathematical and machine learning methods.

## 3 Methodology

### 3.1 Xgboost feature selection

Extreme Gradient Boosting (XGBoost) is a scalable machine learning technique used for tree boosting, which falls within the class of scalable machine learning approaches (24). This method, known as a distributed optimized library for gradient boosting, is capable of analyzing the relevance of each feature in the dataset. It has been demonstrated as a reliable and practical approach in machine learning research (19, 25). In comparison to earlier boosting methods, XGBoost excels at selecting a robust classifier from a set of weaker classifiers. It offers advantages such as effective handling of missing values, avoidance of overfitting, and faster computation times for parallel and distributed models. The primary objective of XGBoost utilizes an optimized gradient descent approach with versatile differentiable loss functions is to employ an optimized gradient descent method with arbitrary differentiable loss functions. This is achieved by incorporating weak learners to minimize the loss function, thus defining and optimizing the overall objective function.

Extreme gradient boosting strives to reduce the objective function in the following manner (as shown in Equation 1).


(1) $$obj\left(\theta \right)=\underset{i}{\sum }L\left({\hat{y}}_{i},y_{i}\right)+\underset{k}{\sum }\Omega \left(f_{k}\right),f_{k}\epsilon \;F$$

The training loss function, denoted as L, quantifies the disparity between the predicted value ${\hat{y}}_{i}$ by the model proposed and actual value of $y_{i}$ . Overfitting is prevented thanks to the regularization function Ω, which estimates the model’s complexity. The set of all possible regression trees is represented by the function f in the functional space *F*. By using parameters and a greedy search method, XGBoost determines the optimal tree structure to minimize the objective function.

### 3.2 SIRVD epidemic model

The SIRVD is derived from the SIR epidemic model (26). This model encompasses dynamics of the virus’s interaction during transmission with the host and classifies individuals into five distinct groups: susceptible, infected, recovered, vaccinated, and deceased (27). The SIRVD expands upon the existing SIR framework by including the new states of vaccinated and deceased. Vaccinated persons are those who have been inoculated against the disease, while deceased individuals are those who have died after becoming sick in the community (28). The ordinary differential equations below represent the mathematical formulation of the SIRVD model (Equations 2–7).


(2) $$\frac{dS_{r}\left(t_{i}\right)}{d\left(t_{i}\right)}=-\frac{\beta l_{r}\left(t_{i}\right)S\left(t_{i}\right)}{N}+\sigma R_{r}\left(t_{i}\right)-\alpha S_{r}\left(t_{i}\right)$$

(3) $$\frac{dI_{r}\left(t_{i}\right)}{d\left(t_{i}\right)}=\frac{\beta l_{r}\left(t_{i}\right)S_{r}\left(t_{i}\right)}{N}-\gamma l_{r}\left(t_{i}\right)-\delta I_{r}\left(t_{i}\right)$$

(4) $$\frac{dR_{r}\left(t_{i}\right)}{d\left(t_{i}\right)}=\gamma I_{r}\left(t_{i}\right)-\sigma R_{r}\left(t_{i}\right)$$

(5) $$\frac{dV_{r}\left(t_{i}\right)}{d\left(t_{i}\right)}=\alpha S_{r}\left(t_{i}\right)$$

(6) $$\frac{dD_{r}\left(t_{i}\right)}{d\left(t_{i}\right)}=\delta I_{r}\left(t_{i}\right)$$

(7) $$N=S_{r}\left(t_{i}\right)+dI_{r}\left(t_{i}\right)+R_{r}\left(t_{i}\right)+V_{r}\left(t_{i}\right)+D_{r}\left(t_{i}\right)$$

where,

β—Infection rate, encompasses the spread of the infection in a susceptible state.
γ—Recovery rate consists of the transferal from the infected to the recovered state.
δ—Rate of death, represents the transferal from the infected to the deceased state.
α—Rate of vaccination consists of the transferal from susceptible to the vaccinated condition.
σ—rate of susceptibility depicts the transferal from recovered to a susceptible state.

It is stated that the transference cycle of the virus is characterized by $\frac{\beta Ir\left(ti\right)Sr\left(ti\right)}{N}$ depicts the number of individuals per unit of time who transmitted from the susceptible individuals ( Sr ) to the infected individuals ( Ir ). The five parameters of the SIRVD epidemic model such as β , γ,δ , α , and σ are considered to be constant, as these are dynamic and thereby, this model neglects their time-dependent characteristics. To predict the growth of the disease trend efficiently and effectively, a time-dependent SIRVD model was proposed, which includes these factors of the SIRVD epidemic model with respect to time ti . The proposed SIRVD epidemic model can reasonably trace the COVID-19 disease transmission and also predicts the future spread of the disease.

### 3.3 SIRVD epidemic time-dependent COVID-19 model

The SIRVD model, which is dependent on time, incorporates five parameters that change over time: the infection rate β, the recovery rate γ, the death rate δ, the vaccination rate α, and the susceptibility rate σ as in Liao et al. (27). These parameters are represented as functions of time, denoted as β( ti ), γ( ti ), δ( ti ), α( ti ), and σ( ti ) (27). The differential equations have been adjusted as follows (Equations 8–12):


(8) $$\frac{dS_{r}\left(t_{i}\right)}{d\left(t_{i}\right)}=-\frac{\beta \left(t_{i}\right)I_{r}\left(t_{i}\right)S_{r}\left(t_{i}\right)}{N}+\sigma \left(t_{i}\right)R_{r}\left(t_{i}\right)-\alpha \left(t_{i}\right)S_{r}\left(t_{i}\right)$$

(9) $$\frac{dI_{r}\left(t_{i}\right)}{d\left(t_{i}\right)}=\frac{\beta \left(t_{i}\right)I_{r}\left(t_{i}\right)S_{r}\left(t_{i}\right)}{N}-\gamma \left(t_{i}\right)I_{r}\left(t_{i}\right)-\delta \left(t_{i}\right)\delta I_{r}\left(t_{i}\right)$$

(10) $$\frac{dR_{r}\left(t_{i}\right)}{d\left(t_{i}\right)}=\gamma \left(t_{i}\right)I_{r}\left(t_{i}\right)-\sigma \left(t_{i}\right)R_{r}\left(t_{i}\right)$$

(11) $$\frac{dV_{r}\left(t_{i}\right)}{d\left(t_{i}\right)}=\alpha \left(t_{i}\right)S_{r}\left(t_{i}\right)$$

(12) $$\frac{dD_{r}\left(t_{i}\right)}{d\left(t_{i}\right)}=\delta \left(t_{i}\right)\delta I_{r}\left(t_{i}\right)$$

*N* is a constant across the population, then the sum of each population’s gain or decrease in the state equals to zero (as shown in Equation 13).


(13) $$\frac{dS_{r}\left(t_{i}\right)}{d\left(t_{i}\right)}+\frac{dI_{r}\left(t_{i}\right)}{d\left(t_{i}\right)}+\frac{dR_{r}\left(t_{i}\right)}{d\left(t_{i}\right)}+\frac{dV_{r}\left(t_{i}\right)}{d\left(t_{i}\right)}+\frac{dD_{r}\left(t_{i}\right)}{d\left(t_{i}\right)}=0$$

Since the COVID-19 data are updated regularly on daily basis, the Equations 8–12 can be changed to differential Equations 14–18.


(14) $$S_{r}\left(t_{i+1}\right)-S_{r}\left(t_{i}\right)=-\frac{\beta \left(t_{i}\right)I_{r}\left(t_{i}\right)S_{r}\left(t_{i}\right)}{N}+\sigma \left(t_{i}\right)R_{r}\left(t_{i}\right)-\alpha \left(t_{i}\right)S_{r}\left(t_{i}\right)$$

(15) $$I_{r}\left(t_{i+1}\right)-I_{r}\left(t_{i}\right)=\frac{\beta \left(t_{i}\right)I_{r}\left(t_{i}\right)S_{r}\left(t_{i}\right)}{N}-\gamma \left(t_{i}\right)I_{r}\left(t_{i}\right)-\delta \left(t_{i}\right)\delta I_{r}\left(t_{i}\right)$$

(16) $$R_{r}\left(t_{i+1}\right)-R_{r}\left(t_{i}\right)=\gamma \left(t_{i}\right)I_{r}\left(t_{i}\right)-\sigma \left(t_{i}\right)R_{r}\left(t_{i}\right)$$

(17) $$V_{r}\left(t_{i+1}\right)-V_{r}\left(t_{i}\right)=\alpha \left(t_{i}\right)S_{r}\left(t_{i}\right)$$

(18) $$D_{r}\left(t_{i+1}\right)-D_{r}\left(t_{i}\right)=\delta \left(t_{i}\right)\delta I_{r}\left(t_{i}\right)$$

Since the human body would create antibodies to the virus, it is believed that the COVID-19 reinfection rate during transmission was approximately equal to zero (29).

Subsequently, the formula of $\gamma \left(t\right)$ can be expressed as (Equations 19–21):


(19) $$\gamma \left(t_{i}\right)=\frac{R_{r}\left(t_{i+1}\right)-R_{r}\left(t_{i}\right)}{I_{r}\left(t_{i}\right)}$$

Similarly,


(20) $$\alpha \left(t_{i}\right)=\frac{S_{r}\left(t_{i+1}\right)-S_{r}\left(t_{i}\right)}{V_{r}\left(t_{i}\right)}$$

(21) $$\delta \left(t_{i}\right)=\frac{D_{r}\left(t_{i+1}\right)-D_{r}\left(t_{i}\right)}{I_{r}\left(t_{i}\right)}$$

Once the rate of death and recovery is computed, add up with Equation 13. Thus, $\beta \left(t_{i}\right)$ , the time dependent parameter can be obtained using Equation 22.


(22) $$\beta \left(ti\right)=\frac{\left(I_{r}\left(t_{i+1}\right)-I_{r}\left(t_{i}\right)+R_{r}\left(t_{i+1}\right)-R_{r}\left(t_{i}\right)+D_{r}\left(t_{i+1}\right)-D_{r}\left(t_{i}\right)\right)\times N}{Ir\left(ti\right)\times Sr\left(ti\right)}$$

### 3.4 Long short-term memory

Long Short Term Memory (LSTM) is a specialized deep learning-based RNN architecture that finds extensive use in practical applications of time series models (30). As a subclass of artificial neural networks, RNNs display dynamic behavior over time due to their interconnected nodes forming a directed graph along a temporal sequence. RNNs can process input sequences of varying lengths by leveraging their internal state or memory. An RNN can be precisely defined as a collection of analogous networks, each transmitting information to a different recipient, enabling them to connect prior knowledge with the current context. However, as this gap widens, RNNs may struggle to learn to establish meaningful relationships in the data, particularly focusing on short-term memory over long-term memory’s influence.

**Figure 1:**
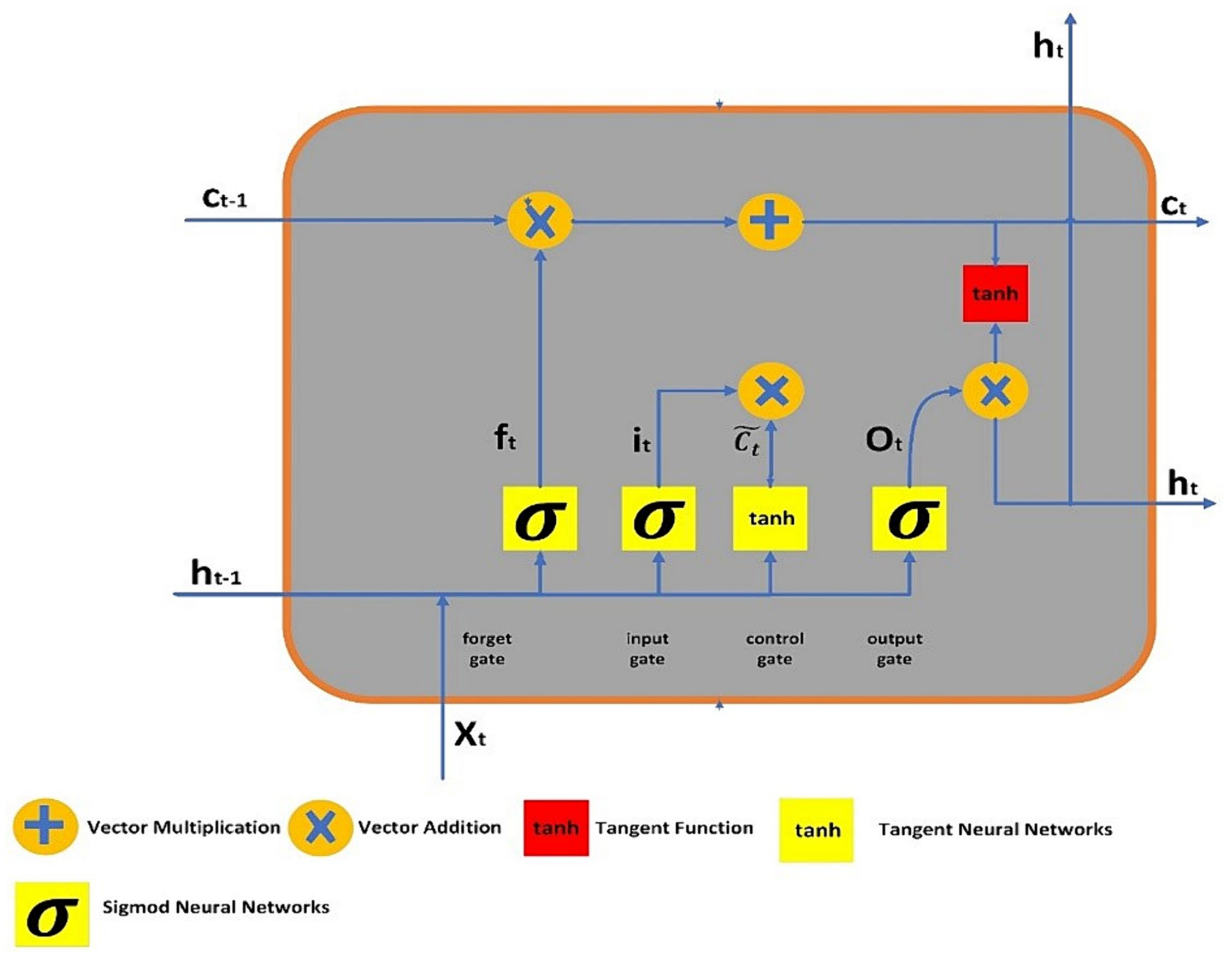
LSTM architecture.

To address the challenges of long-term dependencies, LSTM networks were introduced by Hochreiter and Schmidhuber (30). LSTMs have demonstrated exceptional proficiency in classifying and predicting from time series data. These networks are constructed as chains of replicated modules, each equipped with a unique structure. A typical LSTM unit comprises of memory cell, and three gates say, forget, input, and output. The memory cell possesses the ability of retaining information across extended time intervals, while the three gates discussed earlier controls the information flow in the cell. The output gate determines which value should be stored as the expected output, the input gate decides which additional information to record, and the forget gate selectively discards certain information from the cell state. Figure 1 illustrates the LSTM’s structure, where lines connect entire vectors from one node’s output to another node’s input. The circles represent pointwise operations, while the yellow boxes denote the layers of the previously trained neural network.

The output of LSTM gates, which use sigmoid activation functions to process information, is either 0 or 1. “0” indicates that the gates are blocking everything, and “1” indicates that everything is able to pass past the gates. In the LSTM, the equations of gates are:


(23) $$f_{t}=\sigma \left(w_{f}.\left[a_{t-1},z_{t}\right]+b_{f}\right)$$

(24) $$i_{t}=\sigma \left(w_{i}.\left[a_{t-1},z_{t}\right]+b_{i}\right)$$

(25) $$o_{t}=\sigma \left(w_{o}.\left[a_{t-1},z_{t}\right]+b_{o}\right)$$

From Equations 23–25, *i_t_*, *o_t_* and (30) represents three gates say, forget, input and output. The sigmoid function is denoted by the symbol σ , and x , represents the relevant weight for each LSTM block. $a_{t-1}$ represents the preceding output at t−1 , timestamp, while $z_{t}$ denotes the current input vector at timestamp, t and $b_{x}$ represents bias neurons for gate z . The formulas for the final output, candidate cell state, and cell state are given as follows:


(26) $${\tilde{c}}_{t}=\tanh\left(w_{c}.\left[a_{t+1},z_{t}\right]+b_{c}\right)$$

(27) $$c_{t}=f_{t}*c_{t-1}+i_{c}*c_{t}$$

(28) $$a_{t}=o_{t}*\mathrm{tana}\left(c_{t}\right)$$

From the Equations 26–28, $c_{t}$ and $c_{t-1}$ depicts the current and preceding cell states or memory at t and t−1 timestamps, respectively. The term ${\tilde{c}}_{t}$ expresses to the output of the *tanh* function, which represents the potential cell state at timestamp t . The symbol ∗ denotes element-wise multiplication between vectors.

### 3.5 Proposed XGBoost-SIRVD-LSTM model

**Figure 2:**
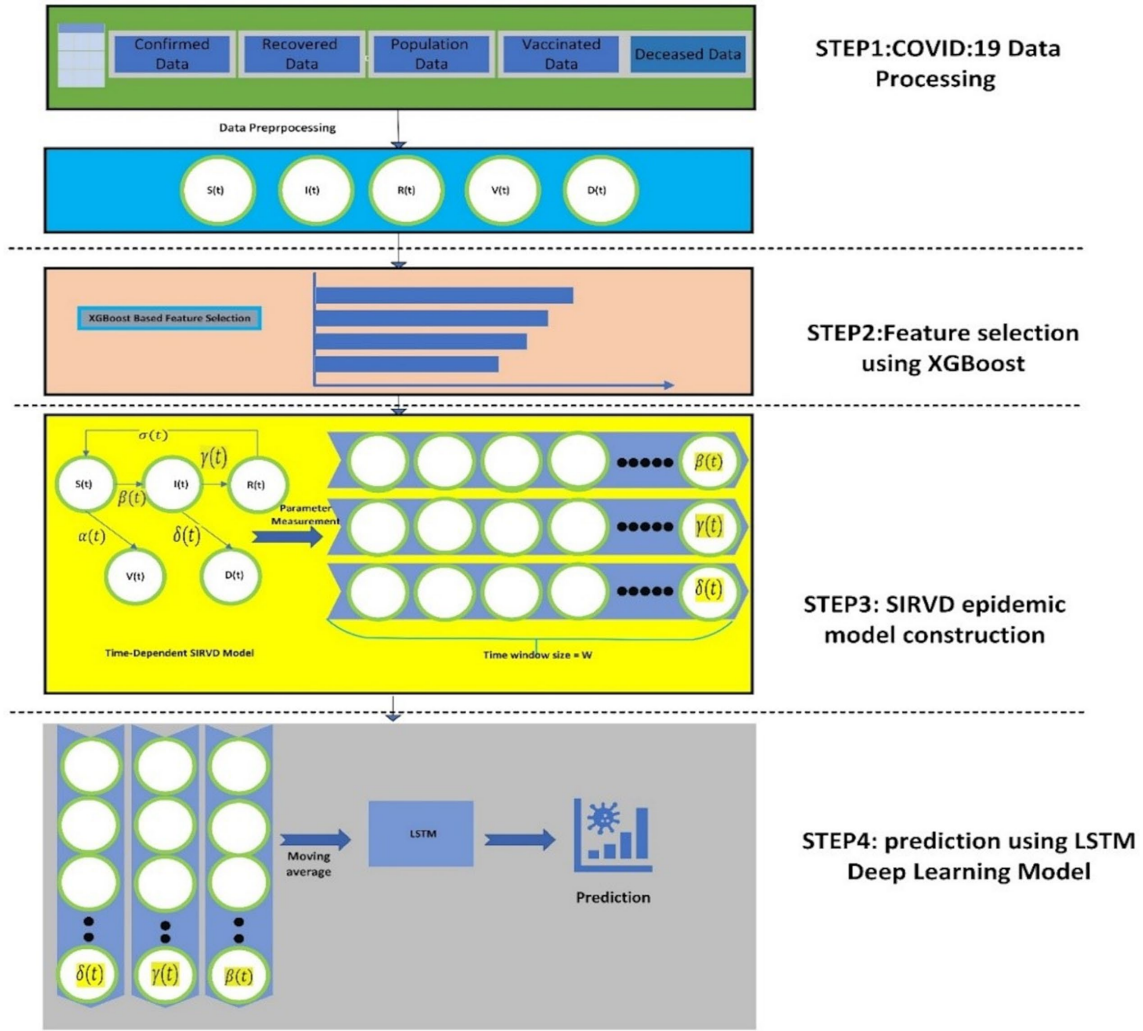
The workflow of the proposed XGBoost-SIRVD-LSTM model for COVID-19 prediction.

Figure 2 shows the suggested model’s workflow details. The proposed XGBoost-SIRVD-LSTM model works in four phases: (1) Data preprocessing, (2) Feature selection using XGBoost feature importance score, (3) SIRVD epidemic model construction, and (4) Prediction using LSTM. This model focuses mainly on the prediction of the recent trends of the epidemic based on the evaluation of the parameter changes in the epidemic. The remainder of this section explains the various stages of the suggested prediction model.

The steps for the proposed XGBoost-SIRVD-LSTM model are as follows:

Input: COVID-19 dataset containing confirmed cases, susceptible cases, recovered cases, deceased cases, and vaccination.
Output: estimating/predicting the COVID-19 infection rate.

*Algorithm steps:*

1. Implement data pre-processing techniques on the COVID-19 dataset.
2. Utilize the Min-Max approach to normalize the dataset.
3. For feature selection, use XGBoost feature importance score.
4. Develop the SIRVD epidemic model with the selected features from step 3.
5. Using step 4, the quantity of COVID-19 infection cases using LSTM are predicted.
6. Evaluate the proposed model using predictive performance metrics.

This section discusses the detailed steps involved in the proposed XGBoost-SIRVD-LSTM model for prediction.

#### 3.5.1 Data pre-processing

The min-max normalization suggested in this paper to pre-process the COVID-19 data. Using below Equation 29, the feature values are normalized between [0, 1].


(29) $$\min-\max\;\mathrm{normalization},y_{i}=\frac{y_{i}-\min}{\max-\min}$$

Where max denotes the highest value and min denotes the least value.

#### 3.5.2 Feature selection using XGBoost feature importance score

The dataset pre-processed after step 1 used for feature optimization in this step. XGBoost feature importance score computed for the optimal selection of features from the COVID-19 dataset (19). Feature importance scores are normalized so that they sum up to 1 across all features. Higher scores indicate more important features relative to others in the dataset. Feature importance scores are useful for feature selection and understanding which features contribute most to the predictions made by the model.

#### 3.5.3 SIRVD epidemic model construction

In this stage, the reduced-feature dataset obtained from step 2 is employed to construct the SIRVD epidemic model. The model incorporates five parameters: (infection) β, (recovery) γ, (death) δ, (vaccination) α, and (susceptibility) σ, which varies over time represented by t (27). The dataset is prepared and formatted according to the specifications of the SIRVD model. The construction of the suggested SIRVD occurs once; dataset has been processed and transformed into the desired format.

#### 3.5.4 Prediction using LSTM

The SIRVD model from step 3 is used for prediction using LSTM in this stage. In this study, single day prediction is computed for predicting the COVID-19 infection, and the model is tested with third, seventh, fourteenth, twenty-first- and twenty-eighth-days’ prediction to evaluate the developed model’s efficacy.

## 4 Results

This section describes the dataset in depth, including the evaluation metrics and efficacy evaluation of the suggested model.

### 4.1 Dataset

Extreme Due to the outbreak of COVID-19, multiple governments worldwide have made public their actions or measures and undertaken real-time data analysis to determine the disease’s up-to-date trends. In this research study, two research data, which are publicly available are collected for experimentation of the proposed model, namely CSSE from Johns Hopkins University (7) and Our World in Data (8). The John Hopkins dataset comprises cumulative cases, including confirmed, recovered, and deceased at a global level. This dataset includes country, province, longitude, latitude, and total affected patients on a specified date as its features.

The data source from Our World in Data includes potential features of interest, namely confirmed and deceased cases, hospitalizations, vaccinations, and testing. The vaccination data obtained from this data source includes various information such as the country name (location), country code (iso_code), date of observation (date), total number of administered doses (total vaccinations), and the count of vaccinated individuals (people_vaccinated). These data, in combination with the data from John Hopkins University, are utilized to implement and assess the proposed model.

### 4.2 Evaluation metrics

The performance of the XGBoost-SIRVD-LSTM model’s performance involves comparing the observed and forecasted values. The evaluation metrics employed in this study include *R*^2^ (determination coefficient) (Equation 32), normalized root mean square error (NRMSE) (Equation 31), root mean square error (RMSE) (Equation 30), and mean absolute percentage error (MAPE) (Equation 33) (31). The validation of the suggested model computed with the following formulas for calculating these metrics.


(30) $$\mathrm{RMSE}=\sqrt{\frac{1}{N}}\overset{N}{\underset{i=1}{\sum }}{\left({\hat{y}}_{i},y_{i}\right)}^{2}$$

(31) $$\mathrm{NRMSE}=\frac{\sqrt{\frac{1}{N}}\sum_{i=1}^{N}{\left({\hat{y}}_{i},y_{i}\right)}^{2}}{\bar{y}}$$

(32) $$R^{2}=\frac{{\left(\sum_{i=1}^{N}\left(\left(x_{i}-\bar{x}\right)\left(y_{i}-\bar{y}\right)\right)\right)}^{2}}{\sum_{i=1}^{N}{\left(x_{i}-\bar{x}\right)}^{2}\times \sum_{i=1}^{N}{\left(y_{i}-\bar{y}\right)}^{2}}$$

(33) $$\mathrm{MAPE}=\frac{1}{N}\overset{N}{\underset{i=1}{\sum }}|\frac{{\hat{y}}_{i-}y_{i}}{y_{i}}|*100\%$$

### 4.3 Performance evaluation

The evaluation of the proposed model involves the utilization of datasets mentioned above. The experiments are conducted using Python, with deep learning libraries: numpy, pandas, keras, and tensorflow. The experimentation is performed on hardware with the following specifications: Intel (R) Core i7-8750H CPU @ 2.20 GHz, 64-bit operating system, RAM of 8.00 GB, and with GPU.

The architecture of deep learning models is determined by their hyper-parameters, which play a crucial role in achieving high-quality models. In this study, the optimal hyper-parameters are determined using a grid search approach. Table 1 presents the hyper-parameters utilized in the developed model. The dataset is split as training and testing sets in the ratio of 70:30 and implemented in training and testing the proposed COVID-19 infection case prediction model. The evaluation metrics described in the equations above are used in this study, and Table 2 compares the single-day prediction results of the developed model with existing models in literature.

**Table 1:** Hyper-parameters for the proposed model.

Hyper-parameter	Test values
Optimizer	{SGD, ADAGRAD, Adam}
Learning rate	{0.01, 0.1, 0.5}
Batch size	{64, 128, 256}
Epochs	{1,000, 2,000, 3,000}

**Table 2:** Results depicting prediction for a single day with the proposed model as well as other models.

Model	R^2^	MAPE	RMSE	NRMSE
Bidirectional LSTM	0.92	3.66	145,200	0.05
GRU	0.96	1.89	89,782	0.03
Stacked LSTM	0.96	2.15	92,065	0.03
Vanilla LSTM	0.92	3.29	151,580	0.05
SIRVD-DL	0.99	0.92	38,519	0.01
XGBoost-SIRVD-LSTM	0.99	0.90	35,025	0.01

**Figure 3:**
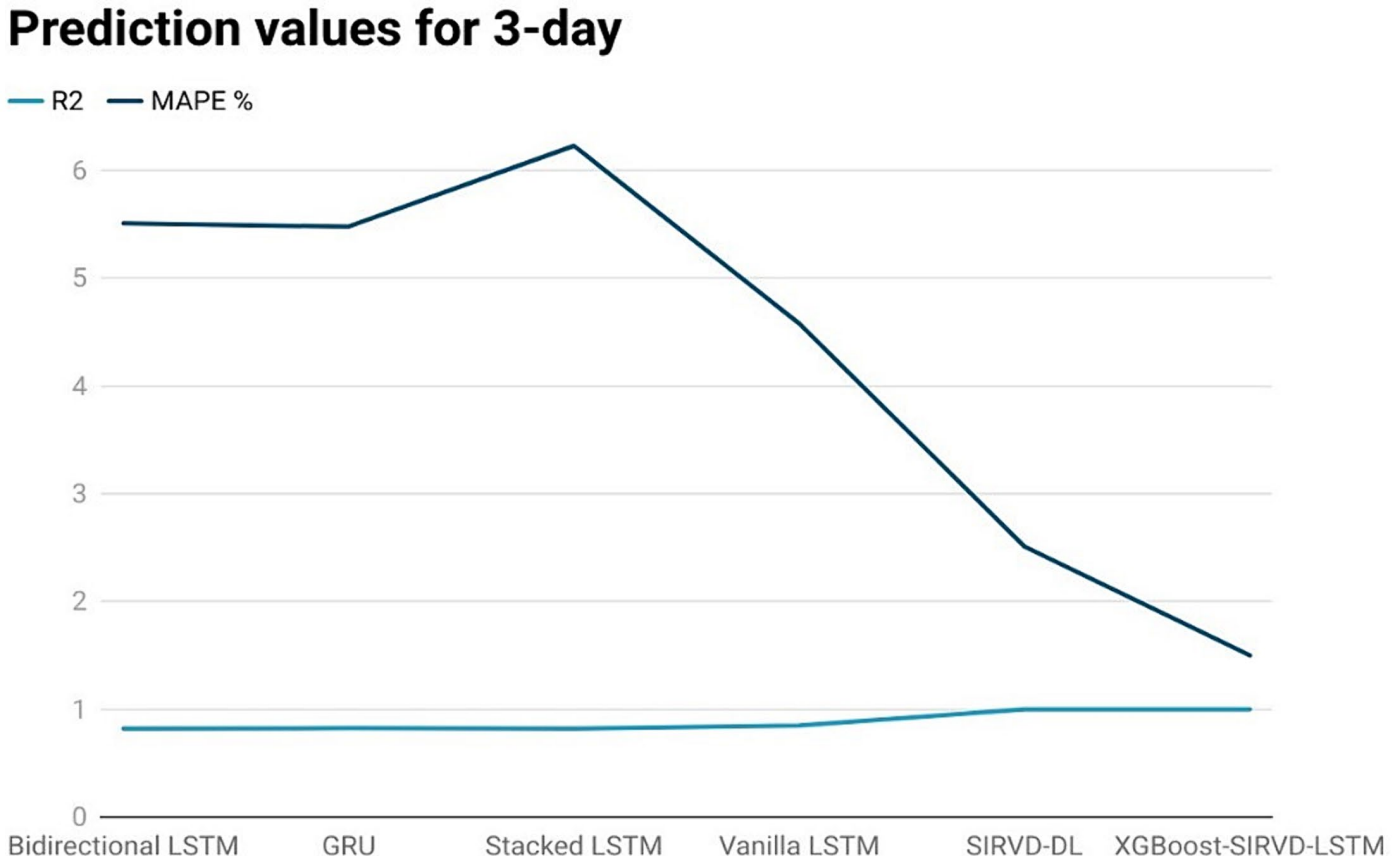
Comparison analysis of prediction results of the suggested model with other models for a 3-day duration.

**Figure 4:**
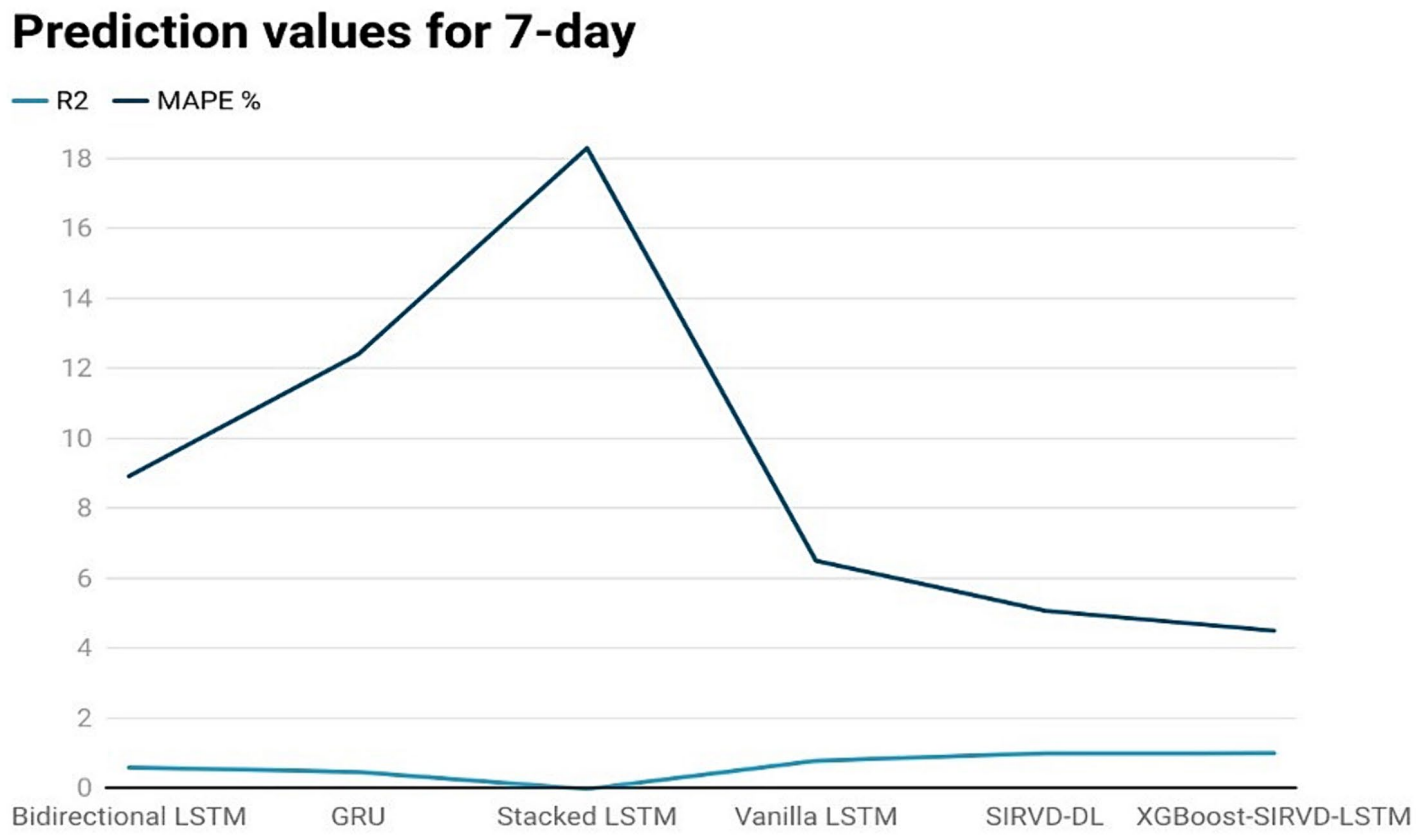
Comparison analysis of prediction results of the suggested model with other models for a 7-day duration.

**Figure 5:**
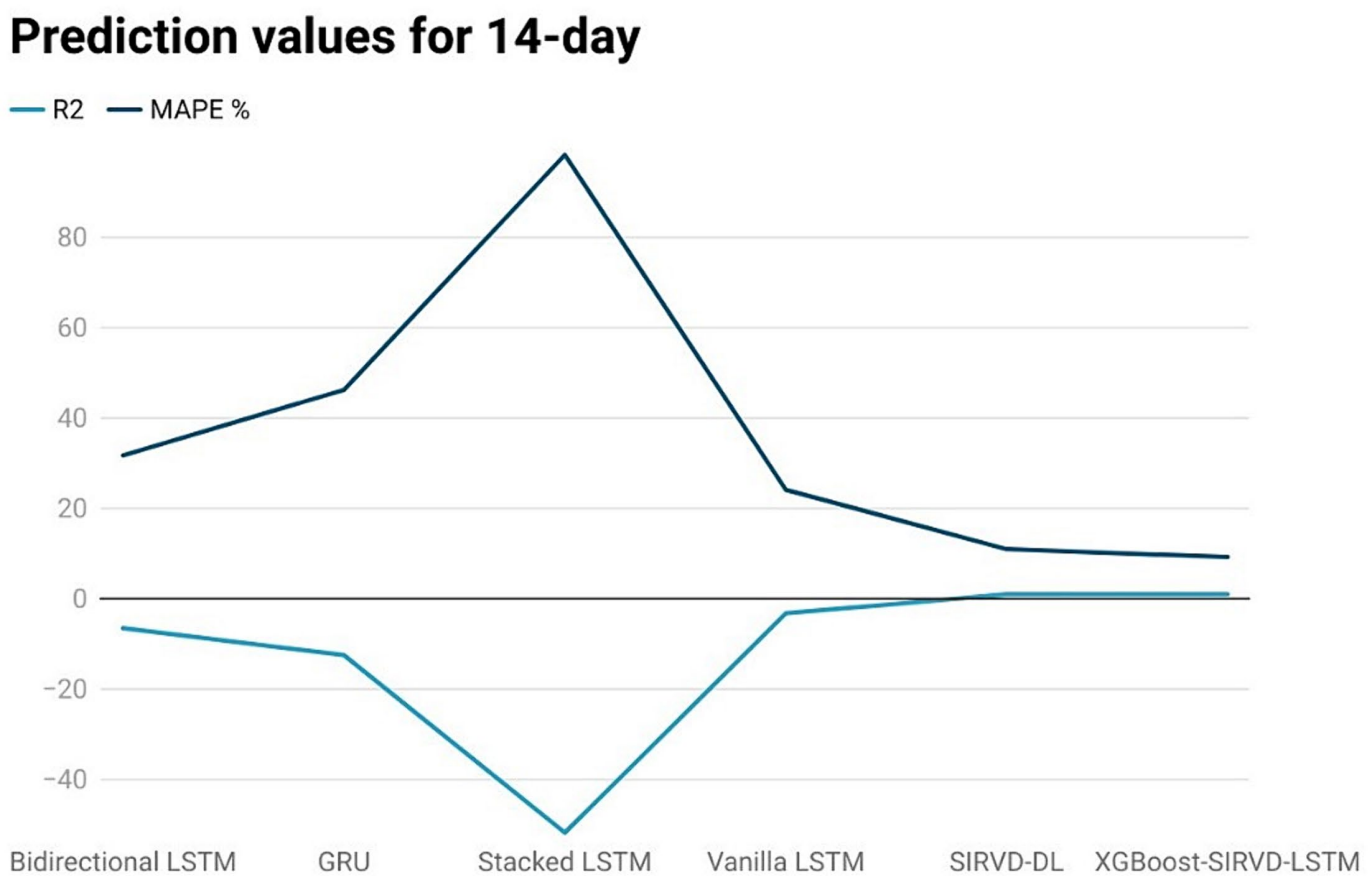
Comparison analysis of prediction results of the suggested model with other models for a 14-day duration.

**Figure 6:**
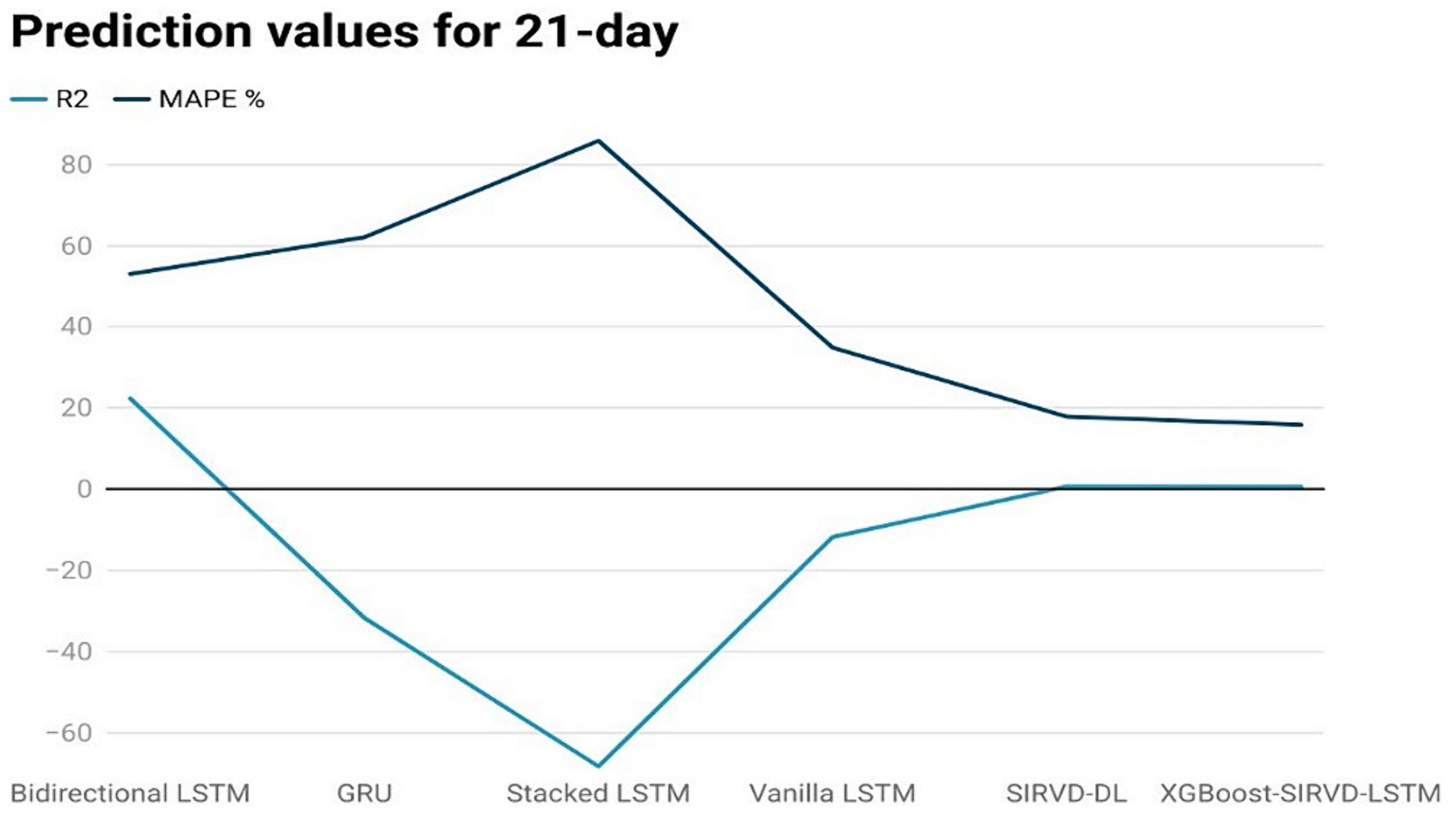
Comparison analysis of prediction results of the suggested model with other models for a 21-day duration.

**Figure 7:**
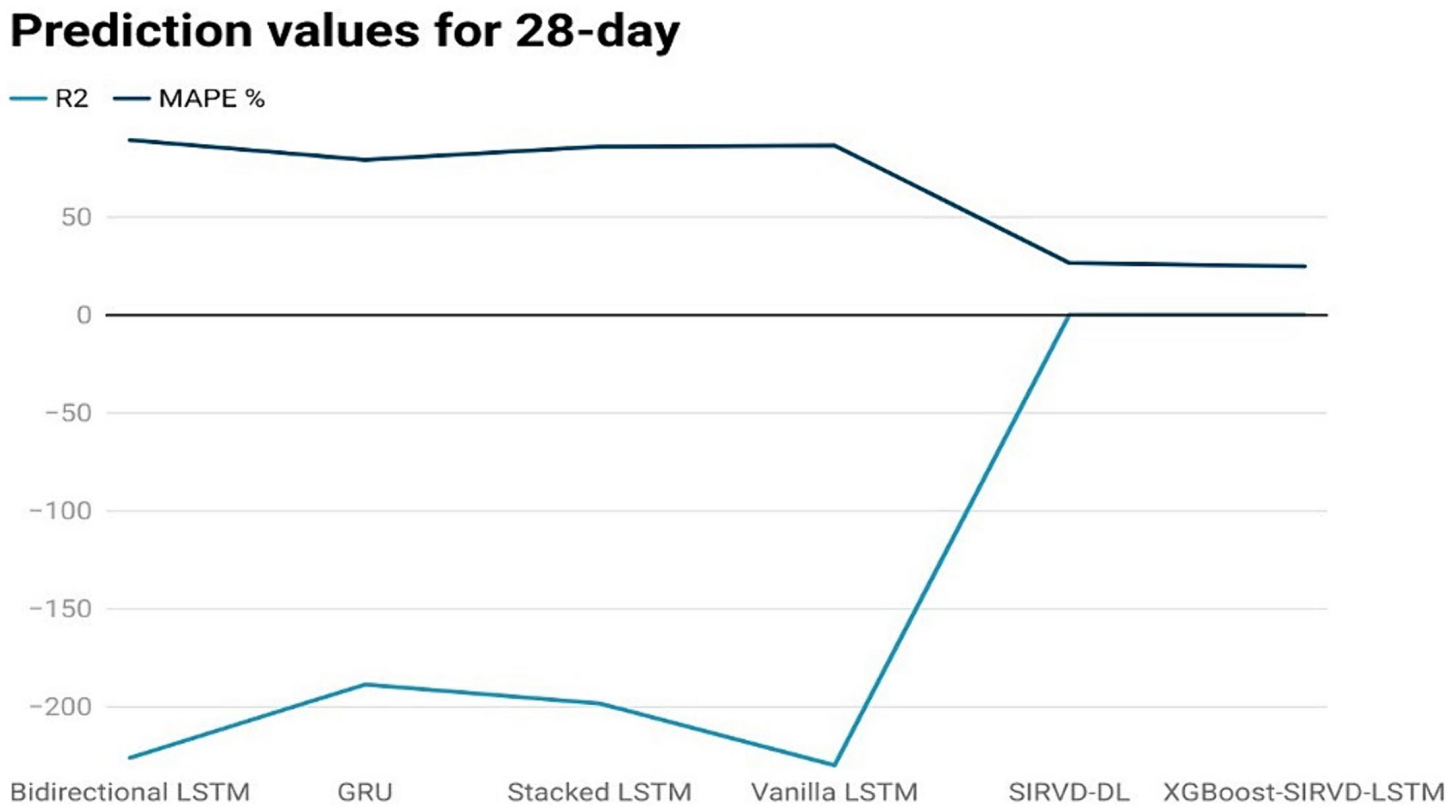
Comparison analysis of prediction results of the suggested model with other models for a 28-day duration.

The effectiveness of the proposed model is assessed by comparing its outcomes with those of existing literature on recurrent deep learning models, including bidirectional LSTM, GRU, Stacked LSTM, Vanilla LSTM, and SIRVD-DL (27). The unique combination of machine learning and mathematical modeling makes the XGBoost-SIRVD-LSTM model better than others. First, using XGBoost for feature selection helps the model find and prioritize key variables, enhancing prediction accuracy. Second, adding the SIRVD model captures COVID-19 transmission dynamics between susceptible, infected, recovered, vaccinated, and deceased populations. Thirdly, LSTM’s sequential data learning allows it to capture COVID-19 temporal patterns and trends. Our comprehensive strategy combines the benefits of each component, resulting in improved prediction accuracy in empirical data. This integrative approach yields more accurate estimates than machine learning or epidemiological models. The experiments were specifically conducted to predict outcomes for the third, seventh, fourteenth, twenty-first, and twenty-eighth days. The experimental results are presented in Figures 3–7. To evaluate the performance of the proposed model, the obtained results are compared to those of other recurrent deep learning models, such as bidirectional LSTM, GRU, stacked LSTM, vanilla LSTM, and SIRVD-DL (27). The experiments were accurately performed to predict outcomes for the third day, seventh day, fourteenth day, twenty-first day, and twenty-eighth day. The experimental findings are displayed in Figures 4–7. Similarly, the proposed model resulted with the *R*^2^ score of 0.999 on the 3-day, 0.997 on the 7-day, 0.956 on the 14-day, 0.64 on the 21-day, and 0.19 on the 28-day. When compared to other models that were taken into consideration for evaluation, the *R*^2^ score grows comparatively as the number of predicting days’ rises, demonstrating the effectiveness of the suggested model. The other models consequently displayed negative values as the number of days increased, indicating that the fitting function’s prediction error was higher than the mean function. As a result, the prediction models’ performance when combined with other models is ineffective.

From the preceding discussion, the contributions of the proposed model can be summarized as follows:

1. A new XGBoost-SIRVD-LSTM model is introduced for predicting COVID-19 infection cases. This model combines XGBoost for feature selection and integrates the SIRVD epidemic model with LSTM for disease prediction.
2. SIRVD-DL and other recurrent deep learning models were used to compare the efficacy of the suggested model.

When compared to previous models, the performance of the proposed XGBoost-SIRVD-LSTM produced improved predictions.

## 5 Conclusion

This research work introduces an innovative model that merges mathematical and machine learning methodologies to forecast the future trajectory of COVID-19. The XGBoost-SIRVD-LSTM model represents a significant advancement in forecasting the course of COVID-19, offering a solution to the critical challenge of precise prediction in the face of a dynamically evolving pandemic. By harmonizing the strengths of XGBoost for feature selection with the SIRVD model’s capacity to track COVID-19 transmission over time, this research provides a comprehensive approach for pandemic forecasting. The dataset is processed using LSTM to provide disease predictions. The model is evaluated using the Our World in Data and CSSE datasets from John Hopkins University. The experimental findings illustrate that the suggested model surpasses alternative deep learning models in terms of performance, exhibiting superior prediction accuracy and precision. These findings suggest that the model proposed will be one of a valuable resource for forecasting the future course of COVID-19. It has the potential to assist governments and public health experts in making informed decisions and formulating effective strategies to combat the pandemic.

Here are some specific potential future research trajectories:

1. Increase the model’s precision and accuracy. More data, more advanced machine learning algorithms, or a mix of the two may be used to achieve this.
2. Improve the model’s usability. This could be achieved by creating a user interface that makes it simple for users to enter data and generate predictions.
3. Predict the efficacy of various therapies using the model. Governments and public health professionals may utilize this information to assist in choosing which actions to prioritize.

## Data Availability

The original contributions presented in the study are included in the article/supplementary material; further inquiries can be directed to the corresponding author.
